# Construction and Validation of a Novel Pyroptosis-Related Four-lncRNA Prognostic Signature Related to Gastric Cancer and Immune Infiltration

**DOI:** 10.3389/fimmu.2022.854785

**Published:** 2022-03-22

**Authors:** Zhengguang Wang, Lei Cao, Sitong Zhou, Jin Lyu, Yang Gao, Ronghua Yang

**Affiliations:** ^1^ Department of Orthopedics, The First Affiliated Hospital of China Medical University, Shenyang, China; ^2^ Department of General Surgery, Tianjin Union Medical Center, Tianjin, China; ^3^ Tianjin Key Laboratory of General Surgery in Construction, Tianjin Union Medical Center, Tianjin, China; ^4^ Department of Dermatology, The First People’s Hospital of Foshan, Foshan, China; ^5^ Department of Pathology, The First People’s Hospital of Foshan, Foshan, China; ^6^ Department of Molecular Pharmacology, School of Medicine, Nankai University, Tianjin, China; ^7^ Department of Burn and Plastic Surgery, Guangzhou First People’s Hospital, School of Medicine, South China University of Technology, Guangzhou, China

**Keywords:** stomach adenocarcinoma, prognostic signature, tumor microenvironment, pyroptosis, immune infiltration

## Abstract

Increasing evidence has demonstrated that pyroptosis, a type of inflammatory programmed cell death, plays an important role in the pathogenesis and progression of gastric cancer. However, it remains unclear whether pyroptosis-related long non-coding RNAs (lncRNAs) can be used to predict the diagnosis and prognosis of gastric adenocarcinoma. This study aimed to evaluate and test the role of the lncRNA signature associated with pyroptosis as a prognostic tool for stomach adenocarcinoma (STAD) and to ascertain their immune value. Relative RNA-sequencing data were extracted from The Cancer Genome Atlas database (TCGA), and data preprocessing was performed for STAD. Pearson correlation analysis was used to determine whether lncRNAs were significantly correlated with pyroptosis based on 23 genes related to pyroptosis. Univariate Cox regression and least absolute shrinkage and selection operator(LASSO) analyses were both adopted to select features and establish the pyroptosis-related lncRNA (PRL) prognostic signature. Kaplan–Meier(KM) survival analysis of the different risk groups was conducted according to the risk scores. We further examined the functional enrichment, tumor microenvironment, and landscape of mutation status among the different risk groups, and these analyses further explained the reasons for the differences in the prediction as well as survival value of the different risk groups. Four lncRNAs, including HAND2-AS1, LINC01354, RP11-276H19.1, and PGM5-AS1, were involved in the PRL signature and used to split STAD patients into two risk groups. Overall survival time(OS) was significantly higher in the low-risk group than in the high-risk group in both the training and validation groups. Functional enrichment analysis was further employed to analyze differentially expressed genes in high- and low-risk groups to identify potential molecular functions and pathways associated with pyroptosis in the gastric cancer microenvironment. Protein-protein interaction (PPI) and Friends analysis identified hub genes that may play a key role in differentially expressed genes in high- and low-risk groups. In addition, there were remarkable discrepancies between the different risk groups in the tumor stage (P < 0.01) and histologic grade (P < 0.05). Furthermore, drug-susceptibility testing indicated potential sensitive chemotherapeutic drugs for each risk group. This study is the first to establish and validate STAD-associated PRLs that can effectively guide the prognosis and the immune microenvironment in STAD patients and provide evidence for the development of molecularly targeted therapies related to pyroptosis.

## 1 Introduction

Gastric cancer (GC), an extremely heterogeneous disease, is the fifth most common cancer and the third leading cause of cancer-related deaths worldwide (1, 2). Stomach adenocarcinoma (STAD), the most common histologic type of gastric cancer, is a rapidly growing, aggressive, and malignant GC that accounts for 95% of all gastric tumors. Although significant clinical advances have been made in the diagnosis and treatment of gastric cancer, the two bottlenecks of low early diagnosis and limited treatment for advanced gastric cancer have resulted in a 5-year survival rate of less than 10% for advanced gastric cancer (3). Therefore, there is an urgent need of discovering novel and reliable biomarkers for the early diagnosis and effective prognosis of STAD.

Pyroptosis is a novel mode of programmed cell death mediated by gasdermin proteins (4). The cleavage of GSDMs *via* the activation of caspase-1/4/5/11 by inflammasomes leads to cell membrane rupture and the release of intracellular pro-inflammatory substances, which triggers a strong inflammatory response in the immune microenvironment (5). As the continued activation of the inflammasome affects tumor progression, pyroptosis is a double-edged sword that plays a dual role in modulating tumor progression. Pyroptosis contributes to the creation of a tumor-suppressive immune microenvironment by liberating inflammatory molecules that can directly destroy cancer cells and galvanize an antitumor immune response (4). In some cases, the induction of pyroptosis can directly kill the tumor cells. Emerging studies have demonstrated the involvement of pyroptosis in cancer growth, differentiation, invasion, and late metastasis, as well as tumor susceptibility to immune drug therapy, among other aspects (6). Pyroptosis-related molecules play an important oncogenic role in gastric cancer progression. Notably, a decreased expression of GSDMD markedly promoted the proliferation of gastric cancers *in vivo* and *in vitro* (7).

Long non-coding RNAs (lncRNAs), with a length of more than 200  nucleotides, are emerging as key regulators of multiple biological processes. Recently, the aberrant expression of lncRNA genes has been suggested to be critical in regulating tumor progression, which is involved in the regulation of tumor proliferation, immune evasion, resistance to cell death, and regional or distant metastasis in STAD (8).

Therefore, lncRNAs are an important breakthrough in deciphering the molecular mechanism of the pyroptosis process, and lncRNA-based studies provide evidence for the development of lncRNAs as therapeutic targets for inducing pyroptosis in gastric cancer. Notably, it has been recently revealed that lncRNAs are crucial modulators of pyroptosis (9). However, the specific role of lncRNAs in the regulation of pyroptosis in the STAD immune system remains largely unknown.

## 2 Material and Methods

### 2.1 Data Acquisition and Preprocessing

TCGA dataset of STAD with 373 samples and clinical information from 406 patients in the UCSC Xena (https://xenabrowser.net/datapages/) was downloaded. Gene mRNA data, copy number variation (CNV) data, Single nucleotide polymorphism (SNP) mutation data, and clinical information for STAD were downloaded from UCSC Xena; the detailed clinical results are presented in the **Supplementary Material**. The data stored by UCSC Xena were standardized by log2 transformation to eliminate the data outline of gene expression within the samples. We applied the annotation file provided by UCSC Xena to re-annotate the expression data and extract all mRNA and lncRNA expression. Human lncRNA information was obtained from the HUGO Gene Nomenclature Committee (HGNC) (10). This study followed the requirements of TCGA publication guidelines. All data used can be found in the TCGA database.

Four expression profiles were obtained during data preprocessing: the mRNA expression profiles of tumor samples (excluding normal samples), the lncRNA expression profiles of tumor samples (excluding normal samples), the mRNA expression profiles of all samples (including normal samples), and the lncRNA expression profiles of all samples (including normal samples).

### 2.2 Collection of Pyroptosis-Related Gene

The whole process of data analysis was depicted in **Supplementary Figure 1**. To screen out the pyroptosis-related gene set, we searched the GeneCards (http://www.genecards.org/), GO BP(http://geneontology.org/), and Reactome databases(https://reactome.org/) with the keyword “pyroptosis”. Then, the jveen package (11) was used to intersect the results from two databases. Results supported by two or more databases were considered as genes associated with cell pyroptosis for further analysis.

### 2.3 Differentially Expressed lncRNA in STAD

Principal component analysis (PCA) analysis was performed using the samples, as indicated. To acquire tumor-associated lncRNAs, differential expression analysis was performed using the limma package (12) on gastric cancer (tumor tissues vs. normal tissues). The screening threshold of differentially expressed lncRNAs was set to a |logFC| ≥ 1.5 and adjusted P-value < 0.01. Differentially expressed lncRNAs were visualized as a heatmap and volcano plot.

### 2.4 Defining Pyroptosis-Related lncRNA (PRL)

To derive the differentially expressed lncRNAs associated with the pyroptosis process, we performed Pearson co-expression analysis of the expression of the pyroptosis-related genes from **Section 2.2** and differential lncRNAs acquired in **Section 2.3** in the tumor samples. We adjusted p-values for multiple testing using the Benjamini–Hochberg (BH) correction and selected the differentially expressed lncRNAs with BH-adjusted P < 0.01 as significantly co-expressed lncRNAs.

### 2.5 Identification of Pyroptosis-Related Prognostic lncRNA

To determine the potential pyroptosis-related prognostic lncRNAs, survival analysis was carried out on these candidate PRLs to determine their effects on STAD patient survival. The lncRNAs with P-values < 0.05 were considered as candidate prognostic lncRNAs and were analyzed further.

### 2.6 Establishment of a Pyroptosis-Related Prognostic lncRNA Model and Prognostic Analysis

The STAD group was randomly divided into a training set and a validation set (sample size, training set: validation set = 2:1). The LASSO method was implemented using the glmSparseNet package (13) in the training dataset(n = 233). The risk score was then constructed using the LASSO regression coefficients and the expression levels of the key lncRNAs. To further validate the prognostic power of the 4-PRLs model for OS prediction, a four-lncRNA signature risk score model was analyzed for the validation dataset (n = 117). For these four lncRNAs, we calculated the Pearson correlation of their respective expression values with those of each pyroptosis-related gene as well as their respective correlations. KM survival curves of each of the four lncRNAs were plotted to evaluate the association between lncRNA expression and OS of the TCGA-STAD patients.

### 2.7 Differential Expression Analyses Between the High- and Low-Risk Groups

Each sample received a risk score according to the novel lncRNA risk score model. Finally, based on the optimal cutoff separation, we divided the TCGA-STAD samples into high- and low-risk groups. To evaluate the differences in the expression profiles of the high- and low-risk subgroups, after PCA, we performed differential analysis between high- and low-risk subgroups using the limma package (12) with a screening threshold of |logFC| > 2 and adjusted P-value < 0.01 to select the differentially expressed genes. The results of the differentially expressed analysis were visualized as a volcano plot and a clustered heat map.

### 2.8 Enrichment and Gene Set Enrichment Analysis (GSEA) Between the High- and Low-Risk Groups

To explore the molecular mechanisms underlying the differences between the high- and low-risk subgroups, we performed GO-Biological Process (GO BP), GO-Cellular Component (GO CC), and GO-Molecular Function (GO MF), and Kyoto Encyclopedia of Genes and Genomes (KEGG) enrichment analyses of the differential genes acquired in **Section 2.7** based on the clusterProfiler package (14) and visualized the enrichment analysis results by selecting the top 15 using bubble plots.

To obtain the expression distribution patterns of the optimal PRLs in the different risk groups, we performed a PCA using scatter plots. Furthermore, GSEA (http://www.gsea-msigdb.org/gsea/index.jsp) was carried out between high- and low-risk groups to evaluate their potential biological functional alterations. Absolute values of normalized enrichment score (NES) >1 and nominal P-values < 0.05 were selected as thresholds of significance.

### 2.9 Gene Set Variation Analysis (GSVA)

GSVA is a nonparametric and unsupervised method that is commonly used to evaluate the variation in pathway activity. GSVA has transformed gene expression data from an expression matrix with individual genes as features to an expression matrix with specific sets of genes. It quantifies the gene enrichment results and facilitates subsequent statistical analysis easily.

GSVA was carried out using the R package “GSVA” (15). We used the limma package (12) for further differential analysis of the GSVA results, with pathways in which |logFC| > 0.15 and adjusted P-value < 0.01 were used to denote significantly differential pathways. The screened significantly differential pathways were visualized as clustered heat maps.

### 2.10 PPI Network and Friends Analysis

Next, we performed a PPI network analysis to identify the key genes among the differentially expressed mRNAs. For this analysis, we uploaded the differential genes obtained in **Section 2.7** to the string database with the parameters of analysis as a string database as default, and then further analyzed and adjusted the network using the Cytoscape (16) software.

Friends analysis hypothesizes that a gene interacts with all other genes in the pathway, and this gene may be more influential and maybe a so-called hub gene. We performed the Friends analysis on the screened differential proteins using the GOSemSim package (17). The top 10 genes from the Friends analysis were visualized and presented as the hub gene for subsequent analysis.

### 2.11 Expression and Prognosis Analyses of Top 10 Hub Genes

Based on the above analysis, we derived key pyroptosis-related mRNAs in STAD. We then performed differential expression, KM survival curves, time-dependent ROC, and co-expression analyses between the top 10 hub genes. Time-dependent ROC curves and area under the ROC curve(AUC) at1-, 3‐ and 5‐years were calculated to assess the predictive performance of the top 10 hub genes using the timeROC package (version 0.4) (https://cran.r-project.org/web/packages/timeROC/index.html) (**Supplementary Figure 2**).

### 2.12 Mutation SNP and Copy Number Variation (CNV) Analysis

To investigate the difference in SNP expression between the high- and low-risk subgroups, we analyzed the downloaded SNP data of STAD using the maftools package (18), in which the mutations of the genes with mutation Top 20 were visualized using waterfall plots with the 10 hub genes derived from **Section 2.12**. Tumor mutation burden (TMB) was defined as the total number of somatic mutations per megabase in each tumor sample. Therefore, we calculated the number of gene mutations in each tumor sample to determine the TMB of each tumor sample. We then performed statistical analysis between groups to determine the TMB in the high- and low-risk subgroups.

We processed the downloaded CNV segment files to obtain the marker files and uploaded the files to the GenePattern Gistic 2.0 module for CNV analysis. The database default was selected for the analysis parameters, and the maftools package (18) was used to visualize the results of CNV analysis.

### 2.13 Estimation of Tumor-Infiltrating Immune Cells in Different Risk Groups

CIBERSORT (19) is an online tool for calculating the relative abundance of immune cells based on the principle of linear support vector regression. To calculate the abundance of 22 types of Tumor-infiltrating immune cells (TIICs) in STAD, the CIBERSORT algorithm with the LM22 gene signature was used. We further compared the difference in the abundance of tumor immune microenvironment cells between the high- and low-risk groups and normal tissues.

### 2.14 Correlation Between Different Risk Groups and Clinical Features

Based on the different high- and low-risk groups, we performed Chi-square tests on the relevant clinical features in different risk groups (histologic grade, new tumor event (after_initial_treatment), pathologic stage, TNM staging, vital status, gender, and race) to analyze and filter the disease processes that may be influenced by high- and low-tumor risk and disease-related events that affect the high- and low-risk subgroups.

### 2.15 The Collection of Patient Tissue Specimens and Quantitative Real-time Polymerase Chain Reaction (qRT-PCR)

A total of 15 human gastric cancer tissues and paired adjacent non-tumor tissues (more than 2.5 cm from the edge of the cancer tissue), were surgically resected and first diagnosed for primary gastric cancer in the Foshan First People’s Hospital between January 2018 and December 2020. Patients who did not receive preoperative chemoradiation treatment were selected for this study. Ethical approval was obtained from the subject review committee of Foshan First People’s Hospital, and all 15 patients informed consent and signed the consent form. Gastric cancer tissues and adjacent tissues were immediately stored in liquid nitrogen after resection for further RNA extraction. Total tissue RNA was isolated using Trizol reagent (15596-026; Invitrogen; Thermo Fisher Scientific, Inc.), followed by cDNA synthesis using Prime-Script RT Master Mix (TaKaRa, Shiga, Japan) for reverse transcription and TaKaRa SYBR^®^ Premix Ex Taq™ (TaKaRa) for qRT-PCR. All primers used in the PCR are listed in **Table 1**. The expression levels of the four risk lncRNAs were normalized to the expression levels of GADPH.

**Table 1:** The sequences used in this study.

Primers	Primers sequence (5′-3′)	
Gene	Forward primer	Reverse primer
*HAND2-AS1*	TTGGGCGATTTTGAAGTGCG	GGTGGAGAGGACTGGTTTCG
LINC01354	GCAATGGTTTGGG CAACTGTAT	GAAAAAGCAAGCTGCCATGAGA
PGM5‐AS1	GACTATGTTGTGAGCCTGCG	AAAAGGGGAGGGGCAATACA
RP11-276H19.1	TCTTCCTGTACCTGCTGAAG	TCACCACGTAAGACATCTGG
GAPDH	GAGTCAACGGATTTGGTCGT	TTGATTTTGGAGGGATCTCG

### 2.16 Drug Sensitivity Analysis

To predict the anticancer drug sensitivity of the different groups, we used the Genomics of Drug Sensitivity in Cancer (GDSC) website to download the anticancer drug dataset and used the oncoPredict package to calculate the relationship between the IC_50_ of different anticancer drugs and different risk groups (20). We screened drugs with a standard mean IC_50_ < 1 for all gastric cancer samples, which were considered to be potent drugs for gastric cancer treatment, and performed statistical tests of drug sensitivity for these drugs in the high- and low-risk subgroups to determine the different levels of response to drugs in patients in different risk groups.

### 2.17 Statistical Analysis

All statistical analyses and plots were performed using R (version 4.0.2). All data are presented as the mean ± standard deviation (SD) derived from at least three separate experiments. Student’s t-test or one-way analysis of variance (ANOVA) was used to evaluate differences among different groups. Otherwise, the correlation was evaluated using the Pearson correlation analysis. The correlations between risk groups and clinical variables were determined using the Chi-square and Student’s t-tests. Survival-related lncRNAs were identified using univariate Cox regression analysis. OS was calculated using the KM method and estimated by the log-rank test. For all statistical methods, statistical significance was set at P < 0.05.

## 3 Results

### 3.1 Screening and Identification of PRLs in STAD

**Figure 1:**
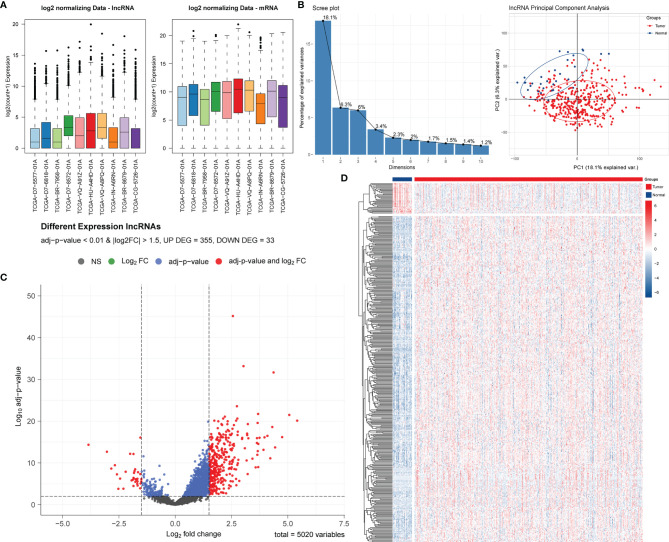
Screening and identification of PRLs in STAD. **(A)** Boxplots of TCGA-STAD data before and after batch effect correction. **(B)** Screen plot of PCA and its results. **(C)** Volcano plot of the differential lncRNA distribution analysis. NS, no significant difference. **(D)** Hierarchical clustering heat map of the differential lncRNA expression analysis.

**Figure 2:**
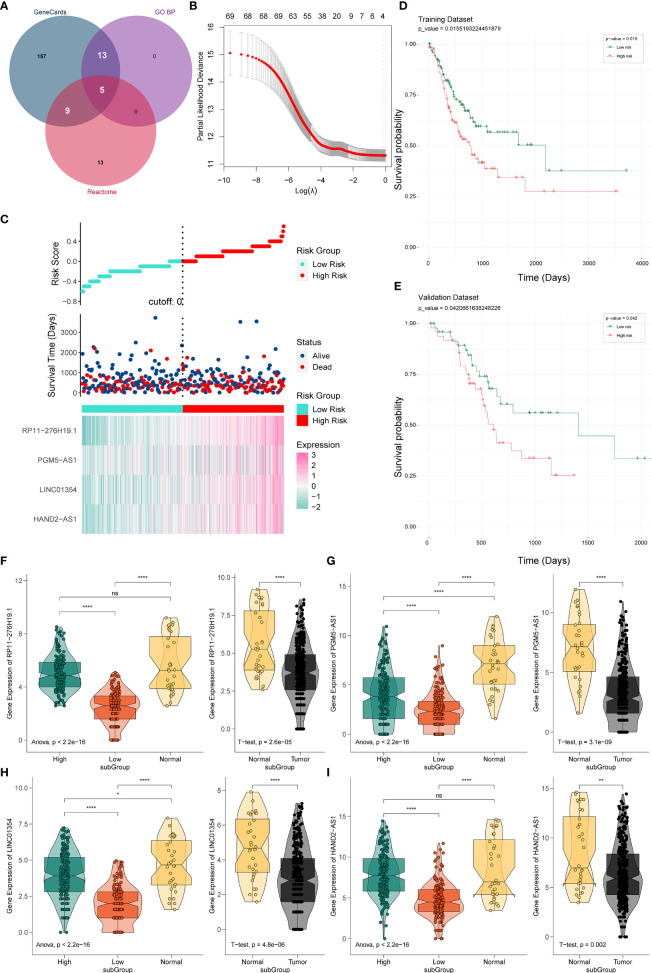
Establishment and verification of the PRL signature. **(A)** Venn diagram of pyroptosis-related genes. **(B)** Predictive modeling using LASSO. **(C)** Construction of the four-PRL prognostic signatures. **(D)** KM survival curves of the four-PRL signature in the training set. **(E)** KM survival curves for the four-PRL signature in the validation set. **(F–I)** The expression level of four-PRLs in high-risk group and low-risk group and in tumor and adjacent normal tissues. ns, no significant difference, *P < 0.05, **P < 0.01, ****P < 0.0001.

After normalization and batch effect adjustment, PCA of lncRNA expression profiles was carried out (**Figures 1A, B** **)**. According to the cut-off criteria of | log2FC | > 1.5 and adjusted P-value < 0.01, there were 388 differentially expressed lncRNAs between cancer and adjacent normal tissues (**Figures 1C, D** **)**. Using jvenn, we found that 27 (9 + 5 + 13) mRNAs were correlated with pyroptosis (**Figure 2A**). Based on the related lncRNA and mRNA expression data, co-expression analysis was performed to estimate the correlation between lncRNA and pyroptosis mRNAs. Next, we carried out lncRNA filtering as described in the Materials and Methods section. According to co-expression analysis, we initially identified 169 significantly positive co-expressed lncRNAs associated with pyroptosis.

### 3.2 Derivation of a PRL Signature for OS Prediction

Prognostic lncRNAs were further screened using univariate Cox regression analysis and LASSO regression analysis. Based on 69 candidate lncRNAs that were highly correlated with OS (P < 0.05) through using univariate Cox regression analysis for selection, then we applied the LASSO method in the training group to establish a PRL signature to evaluate the prognosis of patients with STAD (**Figure 2B**).

The optimal prognostic risk profile of PRL in gastric cancer was composed of four lncRNAs: HAND2-AS1, LINC01354, RP11-276H19.1, and PGM5-AS1 (**Figure 2C**). By combining the expression levels of the four lncRNAs and the derivation of the corresponding regression coefficients from the LASSO analysis, the formula for calculating the risk score for STAD patients obtained was as follows:


Risk score=0.0340×HAND2−AS1+0.0402×LINC01354 −0.0902×PGM5−AS1+0.1370×RP11−276H19.1

Thus, STAD patients were divided into two groups of high and low risk. KM survival analysis showed that the OS time of the low-risk group was significantly higher in the low-risk group than in the high-risk group (training set: P = 0.015, **Figure 2D**; validation set: P = 0.042, **Figure 2E**), indicating that the risk signature of the four PRLs had prognostic value. We then compared the expression levels of four lncRNAs in the different risk groups, as well as in gastric cancer and normal adjacent tissues. Four lncRNAs were found to be expressed at higher levels in the high-risk group than in the low-risk group, while four lncRNAs were expressed at lower levels in gastric cancer tissues than in normal tissues (**Figures 2F‒I**).

### 3.3 Correlation Between Four PRLs Signature With STAD and Pyroptosis Regulators and Survival Validation

**Figure 3:**
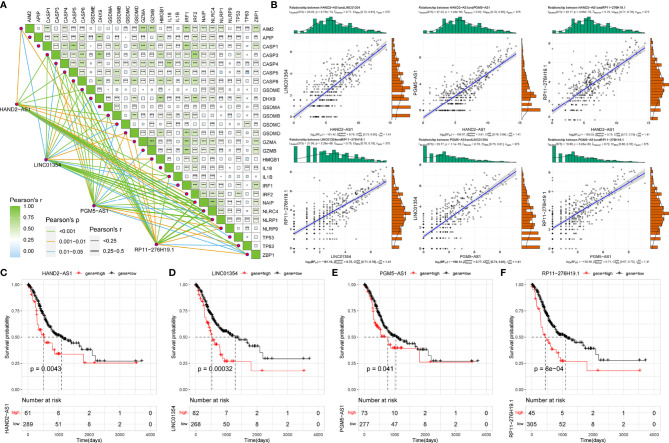
Correlation of four risk PRLs with gastric cancer and pyroptosis. **(A)** Co-expression analysis of the four risk PRLs with pyroptosis-related mRNA. **P < 0.01, ***P < 0.001. **(B)** Co-expression analysis of the four risk PRLs with each other. **(C‒F)** KM survival curves for the four risk lncRNAs.

Using co-expression analysis, we observed a significant positive co-expression relationship between the identified four risk PRLs and pyroptosis-related mRNAs (**Figure 3A**). Significant positive correlations were also observed between the four PRLs (**Figure 3B**). To further investigate the respective prognostic value of the four PRLs in STAD, we performed KM survival analyses to estimate the performance of the four lncRNAs according to their different expression patterns. All four of these PRLs exhibited significant prognostic effects in patients (**Figures 3C‒F**). These results confirmed that the four lncRNAs identified were relevant to pyroptosis of STAD independently.

### 3.4 Differentially Expressed Gene and Functional Enrichment Analysis Between High- and Low-Risk Groups

**Figure 4:**
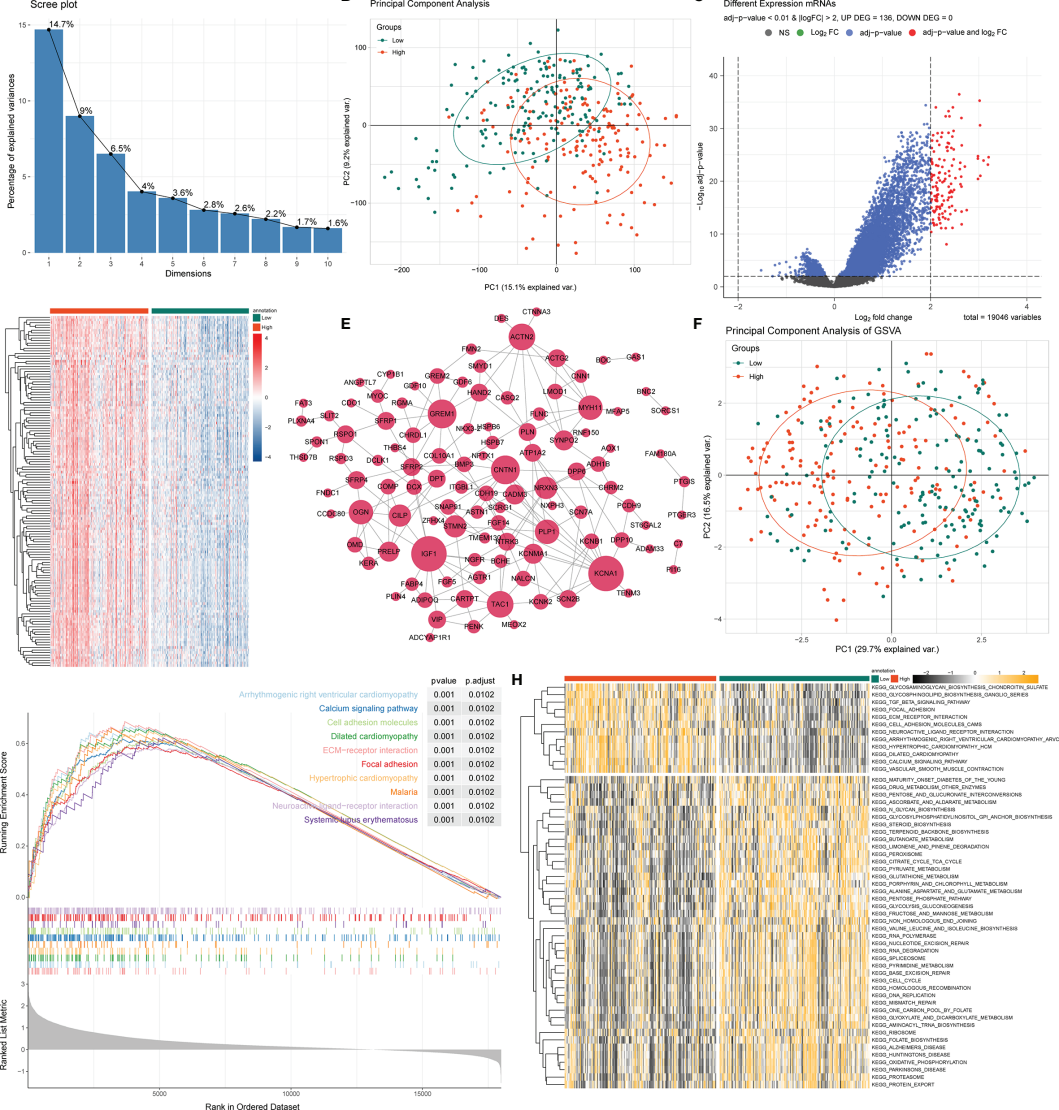
GSEA and GSVA analysis between high- and low-risk groups. **(A)** PCA was applied to the differentially expressed gene analysis of high- and low-risk groups. The results are shown as a screen plot. **(B)** PCA between high- and low-risk groups on all genes. **(C)** Volcano plot of the differential mRNA expression analysis. NS, no significant difference. **(D)** Hierarchical clustering heat map of the differential mRNA expression analysis between different risk groups. **(E)** PPI network analysis of differential mRNAs. **(F)** PCA between high- and low-risk groups on the GSVA. **(G)** The top 10 remarkably enriched pyroptosis-related GSEA pathways between different risk groups with P < 0.001 were selected for plotting. **(H)** Heat maps of gene set variation analysis (GSVA) displayed signaling pathways between different risk groups.

As we stratified the STAD patients into high- and low-risk groups based on their risk scores, differential analysis between the two groups showed significant differences in mRNA expression between the high- and low-risk groups (**Figures 4A, B** **)**. Most of the differentially expressed genes exhibited an upregulated trend in the clustering heatmap and volcano plot between the high- and low-risk groups (**Figures 4C, D** **)**. In addition, we performed a hub gene network analysis of differentially expressed genes using the STRING platform (**Figure 4E**). Furthermore, GSVA was performed to assess potential functional alterations in KEGG pathway activity. PCA revealed distinct grouping between different risk groups (**Figure 4F**). GSVA showed the inflammation-related transforming growth factor (TGF)-β signaling pathway, extracellular matrix (ECM)-receptor interaction, metabolism-related glycosaminoglycan biosynthesis, and glycosphingolipid biosynthesis-ganglio series pathways (**Figure 4G**). We then used GSEA to further predict the potential molecular mechanism differences between the high- and low-risk groups and found that the high-risk group was significantly associated with cell adhesion, ECM-receptor interaction, focal adhesion, calcium signaling pathway, and other biological processes (**Figure 4H**).

### 3.5 Functional Enrichment Analyses of Differentially Expressed Genes Affected by PRLs in STAD

**Figure 5:**
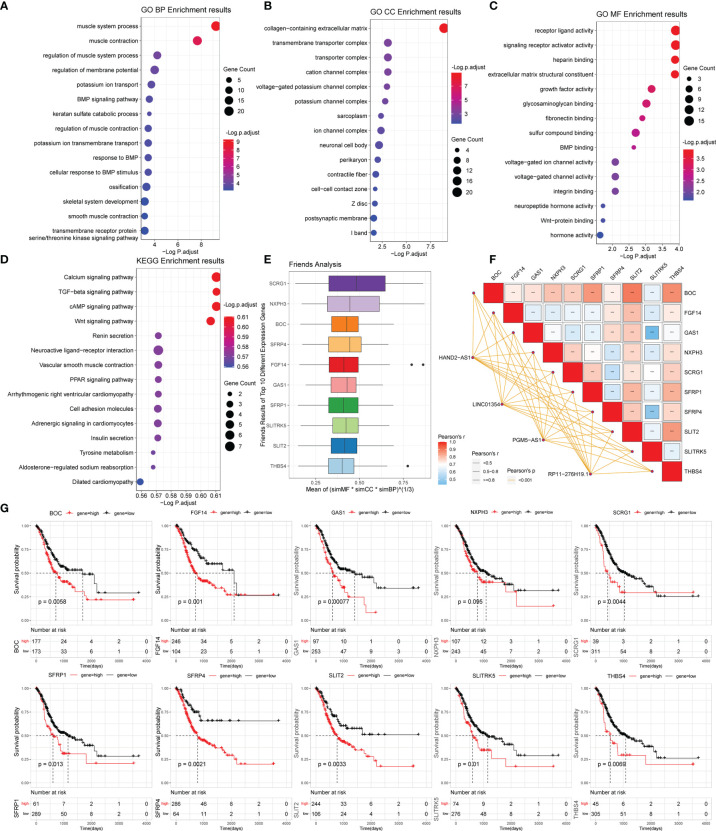
Gene functional enrichment analysis of differentially expressed genes between the high- and low-risk groups. **(A‒C)** GO analysis on the biological processes (BP), cellular components (CC), and molecular functions (MF). **(D)** KEGG enrichment pathway analysis between different risk groups. **(E)** Friends analysis of GO-related genes. **(F)** Correlation analysis between the expression of the 10 hub genes and four PRLs. ***P < 0.001. **(G)** KM survival plotter curve of the ten hub genes.

To analyze the potential biological functions of differentially expressed mRNAs in the high- and low-risk groups, we performed KEGG and GO analyses on the differentially expressed genes (**Figures 5A‒D**). By analyzing the differentially expressed genes between the two groups (all differentially expressed genes were significantly upregulated) (**Figure 4C**), we found that the genes were mainly associated with muscle-related biological processes, such as “muscular system processes” and “muscle contraction,” indicating that the high-risk subgroup was more robust in energy metabolism than the low-risk subgroup. In the following KEGG pathway enrichment analysis, we derived several enrichment results consistent with the previous GSEA and GSVA analyses, such as the calcium signaling pathway and cAMP signaling pathway relevant to energy metabolism and signal transduction, The Wnt and PPAR signaling pathways are relevant to many biological functions, malignancy-associated “cell adhesion molecules,” and “tyrosine metabolism”. These results suggest that there are significant molecular functional differences between the high- and low-risk subgroups of gastric cancer that we obtained from the above analysis. This provides useful information for inferring the potential mechanism of PRLs mediating STAD development.

### 3.6 Identification and Validation of 10 Identified Pyroptosis-Related Hub Genes

**Figure 6:**
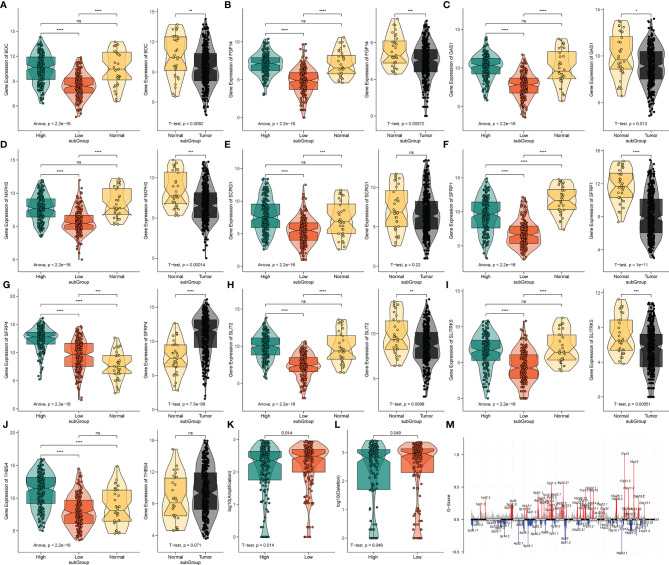
The expression analysis of 10 pyroptosis-related hub genes and CNV analysis. **(A‒J)** The expression profiles of 10 hub genes in TCGA-STAD and normal tissue from the GTEx dataset. ns, no significant difference, *P < 0.05, **P < 0.01, ***P < 0.001, ****P < 0.0001. **(K)** The difference of CNV gain between the different risk groups. **(L)** The difference of CNV loss between the different risk groups. **(M)** The genome-wide gene CNV of STAD.

To further analyze the potential hub genes in the identified pyroptosis-related GO process, we identified the top 10 hub genes using the Friends analysis. Although these genes did not interact too closely with other proteins in the previous PPI network, these 10 hub genes could play a potential role in the pyroptosis-related GO process (**Figure 5E**). Furthermore, there were significant positive co-expression relationships between these 10 hub genes and the four PRLs (**Figure 5F**). To investigate the survival role of 10 hub genes in STAD, correlation analysis of the expression of 10 hub genes and prognosis of gastric cancer patients was performed using the KM survival plotter (**Figure 5G**). Except for NXPH3, the high expression of the other nine genes indicated a poor prognosis in STAD patients (P < 0.05). Next, we investigated the expression levels of these 10 genes in the high- and low-risk groups, as well as in cancer and adjacent normal tissues. The results showed that these 10 pivotal genes tended to be expressed at higher levels in the high-risk group than in the low-risk group, which is further evidence that these genes may be positively regulated by pyroptosis-related risk lncRNAs (**Figures 6A‒J**). Except for SCRG1 and THBS4, the other hub genes were expressed at lower levels in the gastric cancer tissues than in the normal tissues.

### 3.7 Tumor Mutation Status Among Different Risk Groups

**Figure 7:**
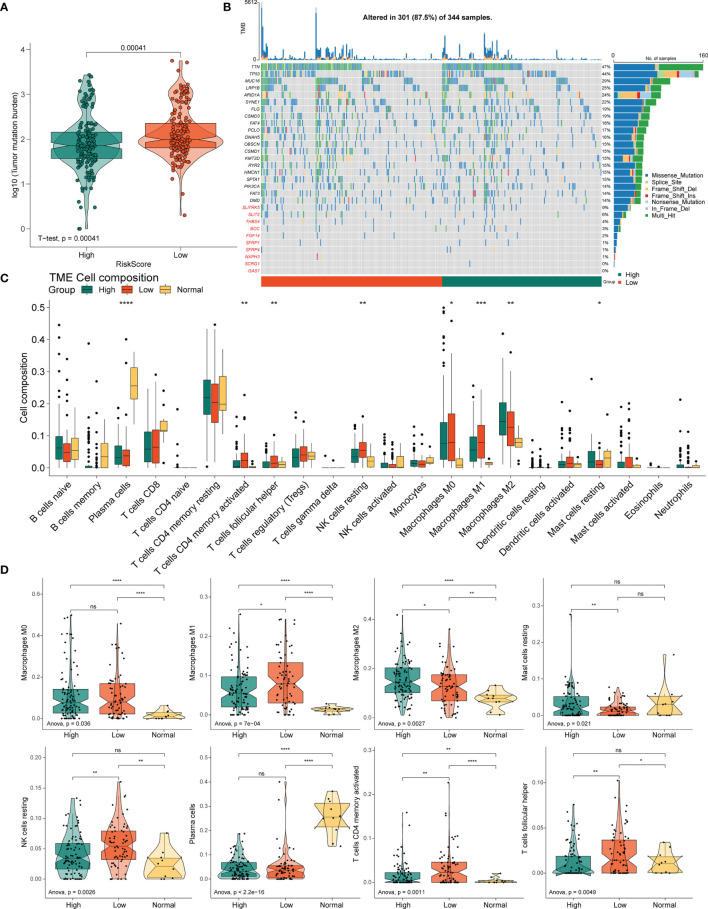
Correlation between the PRLs and tumor immune cells in STAD. **(A)** TMB in the high- and low-risk groups. **(B)** Mutation profile of the top 20 mutation genes and 10 hub genes in STAD. **(C)** Box plot showing the relative abundance of immune cells based on CIBERSORT in the different risk groups and normal tissues. **(D)** The proportion of M0 macrophages, M1 macrophages, M2 macrophages, resting mast cells, resting NK cells, plasma cells, activated resting memory T cells, and follicular helper T cells in the different risk groups and normal tissues. ns, no significant difference, *P < 0.05, **P < 0.01, ***P < < 0.001, ****P < 0.0001.

The variation in the STAD-CNV data demonstrated distinct chromosomal alteration patterns between the high-and low-risk score groups (**Figure 6M**). Next, we analyzed CNV to test whether there were differences between the different risk groups. The low-risk group exhibited elevated frequencies of both copy number amplification and loss compared to the high-risk group (**Figures 6K‒L**). In terms of mutation analysis, we found that the TMB scores were significantly increased in the low-risk groups (**Figure 7A**). Detailed mutation profiles of the top 20 mutated genes and 10 hub genes are shown in the form of a waterfall plot (**Figure 7B**).

### 3.8 Different Risk Groups Exhibited Distinct TIICs and Clinicopathological Features

Next, to determine whether the four novel PRL signatures were related to tumor immunity, we estimated the relationship between the different risk groups and normal tissues and the 22 types of TIICs in STAD using the CIBERSORT algorithm (**Figure 7C**). The low-risk groups contained more M1 macrophages (P < 0.05), resting NK cells (P < 0.01), resting memory CD4(+) T cells (P < 0.01), and follicular helper T cells (P < 0.01) than the high-risk group, thus conferring a significant survival advantage. In contrast, the high-risk group had more monocytes (P < 0.01), M2 macrophages (P < 0.05), resting dendritic cells (DC) (P < 0.001), and resting mast cells (P < 0.05) than the low-risk group (**Figure 7D**).

**Figure 8:**
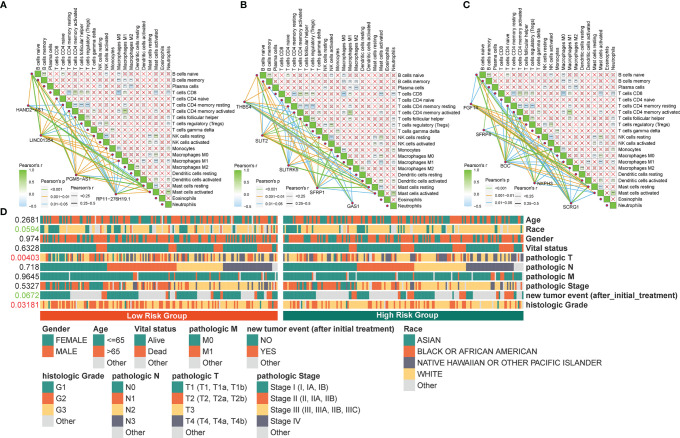
Correlation between the identified pyroptosis-related gene and TIICs and clinicopathological features. **(A‒C)** Correlation of PRLs and PRGs with immune infiltration cells in STAD. **(D)** Correlation between the clinical features and risk scores. ^**^P < 0.01, ^***^P < 0.001.

The four PRLs were positively correlated with the enrichment scores of activated mast cells (**Figures 8A‒C**), and the level of mast cells was upregulated in the high-risk group (**Figure 7D**). Previous studies have demonstrated that mast cells are an independent risk factor for poor prognosis in patients with gastric cancer (21) and that mast cells can indirectly facilitate tumor proliferation and invasion by remodeling the TME composition (22). This partly accounts for the poor prognosis of patients in the high-risk group.

To determine the specificity of the four PRLs in patients with different clinical features, a Chi-square test was used to determine whether the four PRL characteristics affected the clinical characteristics of patients with gastric cancer. The resulting heat map (**Figure 8D**) revealed that there were significant differences between the high- and low-risk groups in terms of the tumor stage (P < 0.01) and histologic grade (P < 0.05).

### 3.9 Validation of the Expression of lncRNAs in Gastric Cancer Tissues

**Figure 9:**
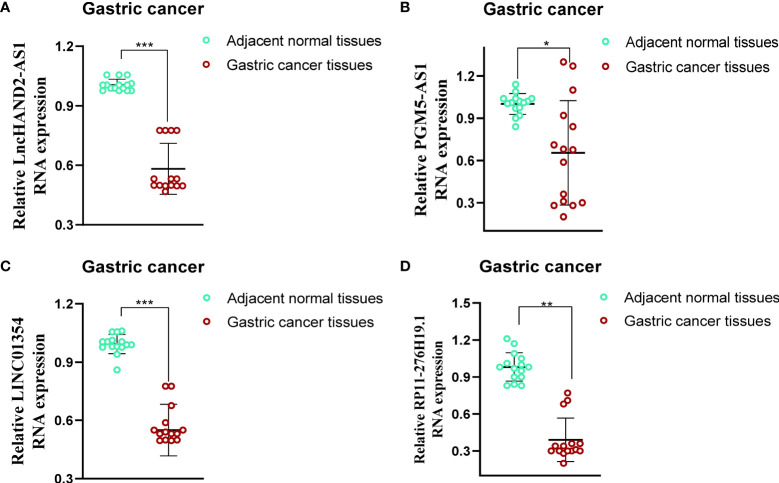
Evaluation of the expression of four PRLs in gastric cancer tissues and adjacent normal tissues (n = 15). **(A)** HAND2-AS1. **(B)** PGM5-AS1. **(C)** LINC01354. **(D)** RP11-276H19.1. ^*^P < 0.05, ^**^P < 0.01, ^***^P < 0.001.

To evaluate the differences in the expression of the four lncRNAs that form the prognostic model in gastric cancer and normal tissues, we employed an unpaired Student’s t-test to detect the expression levels of the four lncRNAs quantified using qRT-PCR. qRT-PCR data from 15 patients showed that the expression of the four lncRNAs was lower and statistically significant in cancer tissues compared to that in paracancerous tissues, which further validated the previous accuracy of our previous bioinformatics analysis (**Figures 9A‒D**).

### 3.10 Drug Rsponses of High- and Low-PRLs Groups in STAD

**Figure 10:**
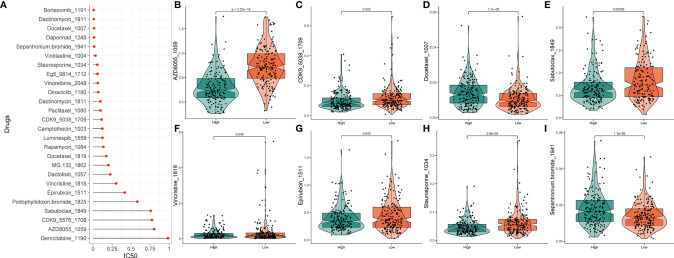
Drug sensitivity analysis. **(A)** IC_50_ training results for drugs with IC_50_ < 1. **(B‒I)** Potential drugs with significant treatment differences in the high- and low-risk subgroups.

Based on the potential role played by lncRNAs in regulating drug sensitivity, we evaluated whether there were differences in the sensitivity of patients in different risk groups to different oncology drugs. The IC_50_ value was estimated for each patient with STAD, according to the predictive model. We identified 26 potential anticancer drugs by screening criteria for IC_50_ < 1, which suggests that these drugs have a strong inhibitory effect on gastric cancer (**Figure 10A**). Eight of these drugs had statistically significant response differences in the different risk groups (P < 0.05) (**Figures 10B‒I**). Apart from docetaxel and sepantronium, most of the chemotherapeutic drugs, including AZD8055, CKD9, staurosporine, vincristine, and epirubicin, showed a lower IC_50_ value in the high-risk groups than in the low-risk groups, which indicated that patients in these groups were more likely to respond well to these chemotherapeutic drugs.

## 4. Discussion

The lack of effective tumor-killing initiators and precise tumor-targeting therapeutic molecules currently hinders the further development of precision therapy for STAD. Recent studies have shown that the targeted therapeutic effect of STAD can be effectively improved by regulating the process of programmed tumor cell death (23). Pyroptosis, a recently discovered mechanism of programmed cell death, has garnered increasing popularity concerning innate immunity, carcinogenesis, and patients’ responses to antitumor treatment (24–26). Pyroptosis occurs in cells infected by pathogens, wherein an inflammatory response and cell lysis are induced within the host body (27). Pyroptosis exhibits two different patterns in tumors. On the one hand, the inflammasome can effectively induce tumor cell death by activating the process of pyroptosis, thus inhibiting the proliferation and invasion of tumor cells (28, 29). For example, low-dose diosbulbin-B (DB) exhibited antitumor effects in gastric cancer by the activation of NLRP3-mediated pyroptotic cell death to enhance the cytotoxic effect of cisplatin-based chemotherapy (25). On the other hand, anomalous pyroptosis favors the tumor microenvironment, which promotes the growth, proliferation, and metastasis of tumor cells (30). The dysregulation of inflammation in the body is a major step in the transformation of normal cells into a carcinogenic lineage. With the stimulation of chronic inflammatory cytokines released by pyroptotic cells in the long term, normal cells can be converted into cancerous cells (31).

Recently, numerous studies have found that the aberrant expression and localization of various lncRNAs in cancer are important contributors to tumor progression (32). They are involved in regulating the process of programmed death in tumors by binding to DNA, miRNAs, and proteins, thus affecting tumor prognosis and therapeutic outcomes. Thus, lncRNAs are increasingly recognized as novel prognostic diagnostic markers and molecularly targeted therapeutic targets for cancers in humans (33). Notably, the overexpression of the lncRNA ADAMTS9-AS2 inhibits gastric cancer progression and promotes cisplatin chemosensitivity by regulating the miR-223-3p/NLRP3 axis-mediated pyroptosis process. This study has revealed the molecular mechanism of chemoresistance to cisplatin in gastric cancer and provided new therapeutic agents for the treatment of gastric cancer in clinical settings (29). Moreover, MEG3 was found to promote cisplatin-induced pyroptosis by promoting the NLRP3/caspase-1/GSDMD axis, implying that MEG3 may be an effective therapeutic target for enhancing breast cancer chemotherapy (34). Ma et al. (35) demonstrated that the inhibition of IncRNARP1-85F18.6 could activate pyroptosis by altering the expression level of ΔNp63 in colorectal cancer. Furthermore, previous studies have demonstrated that lncRNAs modulate direct pyroptosis on inflammasomes. The lncRNA Neat1 promotes inflammasome activation by interacting with NLRP3, NLRC4, and AIM2 inflammasomes in mouse macrophages, thus promoting their assembly and subsequent pro-caspase-1 processing (36). Therefore, establishing a solid PRL signature is crucial for improving prognostic prediction in STAD patients.

In this study, we applied the Pearson correlation method and differential expression analysis to screen 169 potential pyroptosis-related lncRNAs. Next, these lncRNAs were selected to develop a four-PRL signature, including a testing dataset and a validation dataset, demonstrating their sensitivity and specificity. We established a pyroptosis-related prognostic signature based on the expression of four PRLs (HAND2-AS1, LINC01354, RP11-276H19.1, and PGM5-AS1). The high-risk group was found to be mainly enriched in malignant pathways, such as focal adhesion; ECM receptor interaction; TGF-beta signaling pathway; and metabolism-related pathways, such as glycosaminoglycan biosynthesis. These findings indicate a relatively high level of metabolism in individuals at high risk.

Some lncRNAs in the risk model, such as HAND2-AS1, LINC01354, and PGM5-AS1, have been reported to play vital roles in the progression of different cancers, while RP11-276H19.1 was identified for the first time. Patients in the high-risk group had a significantly poorer prognosis than those in the low-risk group. It is worth mentioning that the high expression of each of the four lncRNAs was correlated with poor prognosis in STAD. HAND2-AS1 participates in gastric cancer progression *via* miRNA sponges (37). LINC01315 is upregulated in colorectal cancer (CRC) (38) and osteosarcoma (OS) (39) and has been validated as a novel methylation-related prognostic biomarker in lung adenocarcinoma (LUAD) (40) and laryngeal squamous cell carcinoma (LSCC) (41). PGM5-AS1 is involved in the development of gastric cancer, and PGM5-AS1 has excellent diagnostic value for STAD patients (42). Interestingly, PGM5-AS1 was found to be closely correlated with ferroptosis in LUAD and may represent an oncogene in a risk model for predicting the prognosis of patients with LUAD (43). However, studies on the significance of RP11-276H19.1 in cancer development are currently lacking. All the published studies of PRLs mentioned above have focused on mechanistic investigations in tumor proliferation, invasion, and metastasis; however, their specific roles in pyroptosis are still unclear. An obvious obstacle to the development of antitumor strategies based on pyroptosis is that many cancers appear to either significantly downregulate the expression of GSDM family proteins or express their mutated, non-functional forms, thereby reducing pyroptosis (44). The PRL model developed in this study showed a significant positive correlation with many pyroptosis-related genes, representing a guide for the future development of lncRNA-based pyroptosis-inducing therapeutic regimens.

The Friends analysis of DEGs of different risk groups revealed that SCRG1, NXPH3, BOC, SFRP4, FGF14, GAS1, SFRP1, SLTRK5, SLIT2, and THBS4 are hub regulators in the altered process between different risks. Interestingly, consistent with our PRL prognostic results, the relatively high expression of these hub genes, except NXPH3, indicated a poor prognosis in STAD patients. In particular, SLIT2 is upregulated during allergen stimulation and is involved in the regulation of inflammatory hypersensitivity, demonstrating that SLIT2 may be a potential molecular target for the modulation of pyroptosis (45). Moreover, the high- and low-risk groups exhibited different CNV, TMB, and immune cell infiltration signatures, demonstrating that PRL may play a key modifying role in the regulation of TME and tumor immunity in STAD. In particular, the low-risk group had a higher TMB than the high-risk group, while the low-risk group with a high TMB presented a better prognosis in the STAD cohort, which is consistent with the results of other studies (46, 47). Based on the tumor immune editing hypothesis (48), accompanied by the progression of tumor cells in immunocompetent hosts, fewer immunogenic cancer cells are targeted to evade the antitumor immune response. This may lead to a decrease in immunologically active cell subsets, such as helper T cells, in the tumor microenvironment. Therefore, we postulate that patients in different risk groups will have different responses to immunotherapy. Wu et al. (49) reported that uropathogenic Escherichia coli infection induces pyroptosis in bladder urothelial cells and that they secrete IL-1 and IL-18 in the exosome form, thus promoting the migration process of mast cells, in which mast cells secrete trypsin, further exacerbating the disruption of normal urethral barrier function, thus aggravating urethral infection. These results indicate that the poor prognosis of high-risk patients is derived from a lower TMB and the lower immune responsiveness of the tumor microenvironment, as well as differences in immune cell composition, which together result in the aggressiveness of STAD. Furthermore, we examined the expression levels of these four PRLs by qRT-PCR in 15 pairs of gastric cancer tissues and their paired samples, and the results were consistent with our previous results, increasing the credibility of our study. Finally, we analyzed the differences in sensitivity to chemotherapeutic agents between the high- and low-risk groups and identified potential chemotherapy-sensitive agents. As a result, we found that patients in the high-risk group may be more sensitive to chemotherapy than those in the low-risk group; however, the role of pyroptosis in the difference in sensitivity to chemotherapeutic agents in gastric cancer will need to be confirmed by future studies.

Our study had several limitations. We constructed and optimized four PRLs and validated the expression of four lncRNAs to verify their correlation with the clinical features of STAD. First of all, we initially explored the functional enrichment processes involved in the regulatory network of different risk groups, their specific mechanisms in facilitating pyroptosis still need further investigation to validate our findings. Furthermore, although the model was validated in the TCGA dataset, we further verified the expression of the four PRLs in clinical samples using qRT-PCR. The prediction model constructed in this study still needs to be externally and practically validated to assess its applicability in clinical patients.

This study has three main findings and implications. First, this work represents an initial construction and systematic analysis of a novel 4-PRLs signature to predict the prognosis of STAD patients. Second, we found that the pyroptosis risk score was related to the clinicopathologic features and the alteration of TME, which sheds light on the pyroptosis status and antitumor immune response, thus offering potential molecules for further therapeutic intervention. Third, the modulation of pyroptosis may be a potential therapeutic strategy to improve the outcome of STAD immunotherapy and provide a personalized predictive tool for prognosis and immune response.

## Data Availability Statement

The datasets presented in this study can be found in online repositories. The names of the repository/repositories and accession number(s) can be found in the article/**Supplementary Material**.

## Ethics Statement

The studies involving human participants were reviewed and approved by Ethics Committee of Foshan First People’s Hospital. The patients/participants provided their written informed consent to participate in this study.

## Author Contributions

ZW, RY, and YG conceived and designed this research, obtained the funding and supervised the study. ZW, LC, and SZ performed relevant experiments, the data analysis, and wrote the manuscript. J L collected clinical gastric cancer samples. All authors contributed to the article and approved the submitted version.

## Funding

This study was supported by the National Natural Science Foundation of China (82002913), Natural Science Foundation of Tianjin (Nos. 20JCQNJC01850), and Guangdong Basic and Applied Basic ResearchFoundation (2021B1515120036).

## Conflict of Interest

The authors declare that the research was conducted in the absence of any commercial or financial relationships that could be construed as a potential conflict of interest.

## Publisher’s Note

All claims expressed in this article are solely those of the authors and do not necessarily represent those of their affiliated organizations, or those of the publisher, the editors and the reviewers. Any product that may be evaluated in this article, or claim that may be made by its manufacturer, is not guaranteed or endorsed by the publisher.

## Supplementary Material

Supplementary Figure 1 Flow chart of the construction of pyroptosis-related four-lncRNA prognostic signature.

Supplementary Figure 2 Time-dependent ROC curve of the top 10 hub genes.

## Abbreviations

AUC, Area under the ROC curve; ANOVA, Analysis of variance; BH, Benjamini–Hochberg; CNV, Copy number variation; ECM, Extracellular matrix; FDR, False discovery rate; GSVA, Gene set variation analysis; GSEA, Gene set enrichment analysis; GO, Gene Ontology; GO BP, GO-Biological Process; GO CC, GO-Cellular Component; GO MF, GO-Molecular Function; HR, Hazard ratio; KEGG, Kyoto Encyclopedia of Genes and Genomes; NES, Normalized enrichment score; TCGA, The Cancer Genome Atlas; TGF, The transforming growth factor; TIICs, Tumor-infiltrating immune cells; TME, Tumor microenvironment; LncRNA, Long non-coding RNA; Lasso, Least absolute shrinkage and selection operator; OS, Overall survival; PCC, Pearson correlation coefficient; PRLs, Pyroptosis-related long non-coding RNAs; PRGs,Pyroptosis-related genes; PCA, Principal components analysis; ROC, Receiver-operating characteristic; STAD, Stomach adenocarcinoma.
