# Supplementary figures and images for: Construction and Validation of a Novel Pyroptosis-Related Four-lncRNA Prognostic Signature Related to Gastric Cancer and Immune Infiltration
Source: Front Immunol. 2022 Mar 22;13:854785. doi: 10.3389/fimmu.2022.854785 (PMC8980360; doi:10.3389/fimmu.2022.854785)

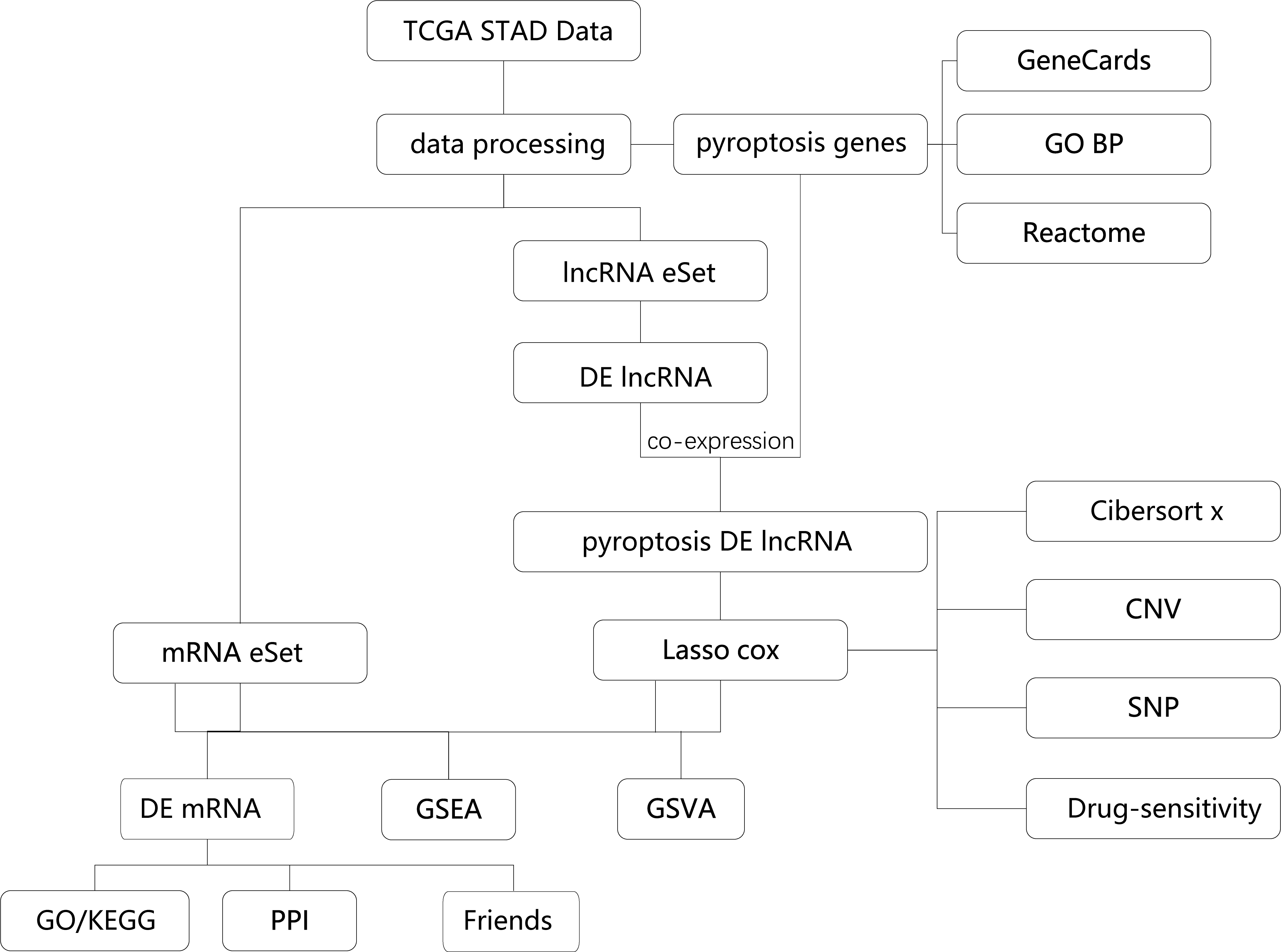

Supplement: Supplementary Figure 1 — Flow chart of the construction of pyroptosis-related four-lncRNA prognostic signature. [file Image_1.tif]

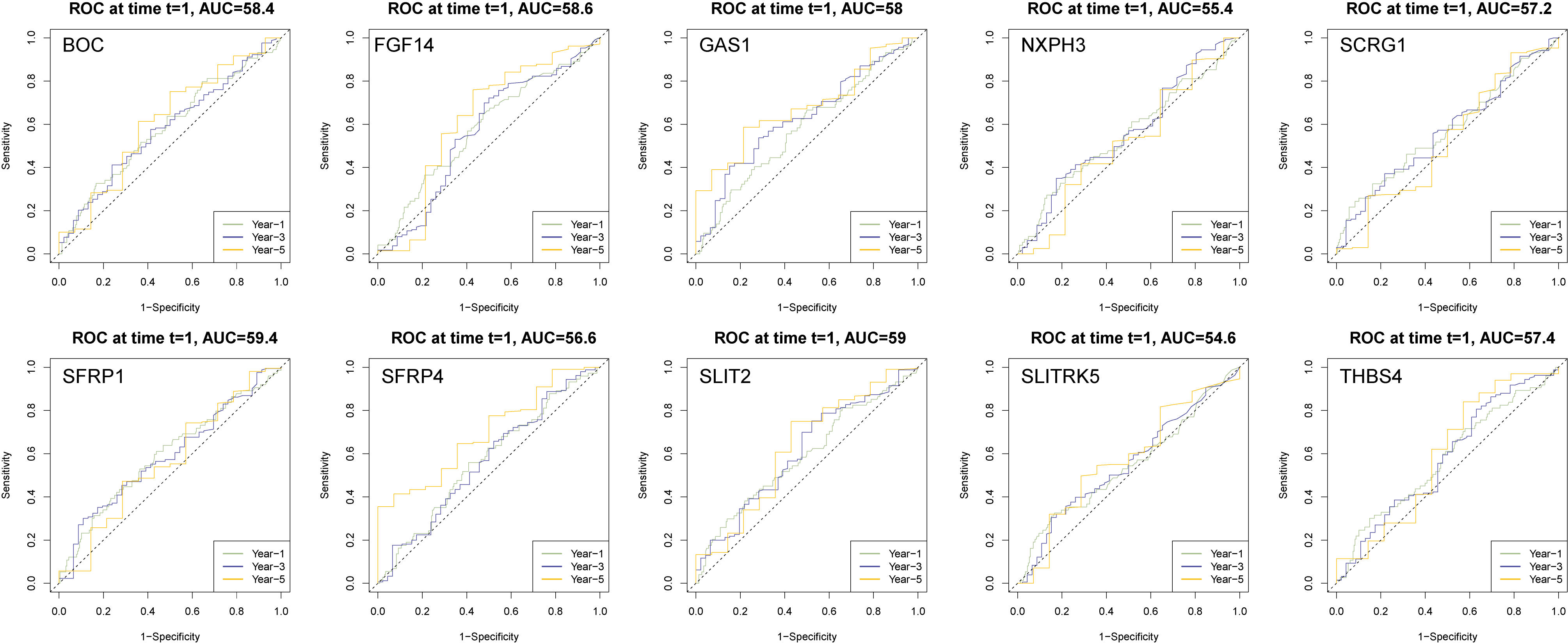

Supplement: Supplementary Figure 2 — Time-dependent ROC curve of the top 10 hub genes. [file Image_2.tif]
